# Body donation under Italy's recent legal reforms: A cross‐sectional study of attitudes, beliefs, and educational gaps among medical students and faculty

**DOI:** 10.1002/ase.70084

**Published:** 2025-07-06

**Authors:** Mariangela V. Puci, Maria A. Sotgiu, Narcisa Muresu, Laura Saderi, Veronica Moretti, Stefano Ratti, Andrea F. Piana, Andrea Montella, Giovanni Sotgiu

**Affiliations:** ^1^ Clinical Epidemiology and Medical Statistics Unit, Department of Medicine, Surgery and Pharmacy University of Sassari Sassari Italy; ^2^ Department of Biomedical Sciences University of Sassari Sassari Italy; ^3^ Department of Humanities and Social Science University of Sassari Sassari Italy; ^4^ Department of Sociology and Business Law University of Bologna Bologna Italy; ^5^ Cellular Signalling Laboratory, Anatomy Centre, Department of Biomedical and NeuroMotor Sciences (DIBINEM) University of Bologna Bologna Italy; ^6^ Department of Medicine, Surgery and Pharmacy University of Sassari Sassari Italy

**Keywords:** academic survey, anatomical dissection, body donation, Italian anatomy laws, medical education, students' attitude

## Abstract

Postmortem body donation (BD) plays a central role in medical education and scientific research. Sociocultural, religious, and legal factors can influence attitudes toward BD. In Italy, BD programs are in early development, and recent legislation (Law 10/2020) regulating body donation came into effect in 2021. Although international surveys have explored attitudes toward BD, data specific to the Italian context remain limited. This study provides initial insights into attitudes and willingness to donate among medical academics and students at an Italian university. A cross‐sectional study was conducted between February and March 2023 using an online questionnaire. The survey collected data on attitudes, dissection experience, sociodemographic, and academic background. A multivariate logistic regression model was implemented to evaluate factors associated with willingness to donate. Of the 2273 individuals invited, 434 completed the questionnaire (19.4% response rate, 70% female, 88% students). Overall, 72.8% were willing to donate. Knowing organ donors was associated with higher willingness, whereas religious beliefs were negatively associated. Only 32% of participants were aware of the national law regulating BD. This study highlights the predominance of positive attitudes toward BD within a healthcare‐oriented academic population and the significant influence of religion and personal experience. The limited awareness of Law 10/2020 underscores the need for targeted educational efforts, even within expert communities. These findings support future initiatives aimed at improving awareness, shaping national policy, and contributing to the global discourse on ethical and practical aspects of body donation.

## Graphical abstract

Multivariate analysis: factors associated with willingness to body donation.

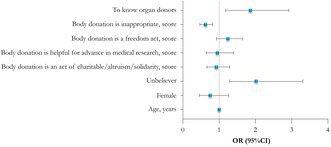

## INTRODUCTION

Human dissection is a key component of the anatomy curriculum.^1^, ^2^, ^3^ Despite ongoing debate on optimal teaching methods,^4^ anatomical dissection is usually preferred by anatomists and students as an effective learning environment that fulfills all anatomy learning objectives, including the development of professional skills and balancing of emotional responses in future doctors.^5^, ^6^ It improves examination performance of medical students,^2^, ^7^, ^8^, ^9^ and the direct visualization of anatomical structures enables students to better appreciate anatomical details and variability compared to virtual 2D or 3D images (McBride & Drake, 2015).^10^, ^11^, ^12^ Additionally, it affords the learner opportunities to reflect on death and dying^13^ and to recognize the educational value of a deceased body as a “first patient” or “silent teacher”.^10^, ^14^, ^15^, ^16^ This perspective emphasizes the importance of being professionally responsible for donors as well as for future patients.

Historically, body procurement for anatomical study has relied on unclaimed bodies or, rarely, on bodies of executed criminals.^7^, ^17^, ^18^, ^19^, ^20^, ^21^, ^22^ In contrast, many countries—including Austria, France, Germany, Malta, the Netherlands, Portugal, Spain, Switzerland, and UK—have developed body donation programs based on voluntary donor consent, providing an ethical alternative.^20^, ^23^, ^24^, ^25^, ^26^, ^27^, ^28^ In 2012, the International Federation of Associations of Anatomists (IFAA) recommended the use of voluntarily donated bodies.^29^ It underscored the ethical role of the informed consent, the non‐commercial nature, and the respect for donors.^30^ However, several countries have not yet implemented organized body donation programs and continue to rely on unclaimed bodies; in some cases, institutions even import preserved bodies from abroad.^17^, ^20^ This practice raises ethical concerns about the commercialization of human tissue through international importation, which may risk creating a perception of trade in human body parts.^31^ In the United States, the Uniform Anatomical Gift Act (UAGA, 1968) regulates body donation, although procedures vary among institutions and states.^32^ Similarly, Europe exhibits significant variability in legal and ethical frameworks for body bequests, as highlighted by the Trans‐European Pedagogic Anatomical Research Group (TEPARG).^25^, ^27^, ^33^

In Italy, body donation programs are in an early stage of development. Law No. 10/2020 titled “*Rules on the disposition of one's own body and post‐mortem tissues for study purposes, training and scientific research*”, was recently approved to regulate the use of the body and/or tissues of a deceased individual for study, training, and research purposes. This legislation establishes strict criteria for body donation, including the requirement of informed consent from living donors, appropriate management of donors' bodies, and identification of national reference centers.^34^, ^35^, ^36^, ^37^ Previously, body donation was governed by Article 32 of the Regio Decreto no. 1592 of August 31, 1933 and the Presidential Decree no. 285/1990, which allowed the use of unclaimed bodies based on the presumed consent (“silent approval”). However, the Italian National Bioethics Committee declared this approach ethically unacceptable due to the absence of explicit consent.^38^ In 2017, Law No. 219 on end‐of‐life decisions was enacted, granting individuals the right to make autonomous choices regarding all end‐of‐life matters, including body donation.^39^, ^40^ Building upon this framework, Law No. 10/2020 allows individuals to document their decision to donate their bodies prior to death and promotes the development of a culture of body donation, establishing informed, explicit, and documented consent as the ethical cornerstone of body donation. The law stipulates that the individual's will is binding, and family members cannot override this decision.

Additionally, the law provides for the designation of authorized centers for the ethical management and conservation of donated bodies and identifies healthcare professionals as appropriate figures to support public awareness and provide education about body donation.^35^ While the law protects the individual's decision, it is essential to educate potential donors about the need to communicate their intentions clearly with their family, to ensure there is no confusion or potential for misunderstandings at the time of death.

By moving away from presumed consent and establishing a clear legal pathway for voluntary donation, Law No. 10/2020 has the potential to significantly impact the availability of bodies for anatomical dissection—a learning method strongly supported in the literature for its educational benefits and its role in developing professional skills and ethical considerations among future doctors.

However, despite this promising legal framework, the practical implementation of these goals remains limited. For example, the Azienda Ospedaliero‐Universitaria of Sassari, Italy is currently one of the few national centers authorized to conserve and utilize postmortem bodies and tissues for research, education, and training purposes [Art. 3, Decreto del Ministro della Salute, 24 aprile 9 2024]. Despite growing interest among prospective donors, a shortage of donated bodies persists, hindering the full implementation of anatomical dissection courses in university curricula.

Unlike organ donation, which has been culturally accepted and legally regulated in Italy since the introduction of Law No. 91/1999,^41^ body donation remains relatively unfamiliar among the general public, including medical students who could play a critical role in raising awareness.^42^ Findings from a recent survey indicate that a substantial proportion of Italian medical students are unaware of the new legislation.^43^

A critical factor influencing body donor availability is the attitude of potential donors and their families. Multiple factors shape these attitudes, including altruism—often cited as a key motivator^42^, ^44^, ^45^, ^46^—as well as cultural, religious, educational, and economic influences.^47^ These factors may also impact individuals who benefit from anatomical donation, such as future healthcare professionals.^48^ For example, a study conducted in Australia involving over 2000 university students found that personal concerns and religious beliefs significantly influenced attitudes toward body donation.^49^

Although attitudes and willingness toward body donation have been explored in various countries, the Italian context remains underexamined, particularly in light of the recent introduction of Law No. 10/2020. Despite this new legislation, data on public awareness and attitudes within Italy are still limited.

Given the influential role of healthcare students and academics in shaping public opinion and supporting donation programs, assessing their views is critical. This descriptive cross‐sectional study examines the attitudes, beliefs, and willingness of students and academics at the University of Sassari to donate their bodies. It also investigates the impact of demographic, social, and cultural factors on donation willingness and evaluates participants' knowledge of the new legal framework.

## METHODS

### Study design and participants

A cross‐sectional study, web‐based survey, was conducted among students and academic staff of the School of Medicine at the University of Sassari, Italy.

The inclusion criteria were defined as follows: Bachelor and Master of Science (i.e., nursing, midwifery, imaging, and radiotherapy techniques, physiotherapy, environment and workplace prevention techniques, biomedical laboratory techniques courses) medical specializations, and PhD programs students; professors, researchers, and research fellows (classified as “academic staff”). The only exclusion criterion was declining to participate in the survey.

Subjects were recruited by official university mailing list and voluntarily participated. Participants were granted anonymity and confidentiality. The University Ethical Committee approved the study protocol (n. 8506, February 2, 2023). No form of compensation was provided for people who participated to the survey.

### Data collection and questionnaire

An ad hoc questionnaire was designed based on previous surveys^43^, ^50^, ^51^ and administered (in Italian language) using the “Form” Google platform from February 22 to March 16, 2023. The questionnaire consisted of two sections, with all questions compulsory (only one response for each item). The first section included sociodemographic characteristics (i.e., age, sex, profession, course degree, personal and family religious beliefs). The second one focused on attitudes toward body donation and included questions about participants' knowledge of national legislation, their opinions on body dissection practices, and voluntary social activities. Attitudes toward body donation were measured using a Likert scale, where participants could express their level of agreement or disagreement on a scale ranging from 1 (completely disagree) to 5 (completely agree) (Table S1).

A pretest was performed to assess its reliability and improve the items' quality by a pilot study that enrolled 30 participants.

### Statistical analysis

A sample size of 317 subjects was estimated assuming a proportion of willingness to donate their body of 29%,^51^ a 95% confidence level, and a margin error of 5%.^52^

Descriptive statistics included measures of central tendency (mean or median) and variability (standard deviation—SD or interquartile range—IQR), and frequencies (absolute and percentages), as appropriate. Comparison of qualitative variables was performed using either chi‐squared or Fisher exact test, whereas for quantitative ones Mann–Whitney test was used. Multivariate logistic regression evaluated the potential association between willingness to body donation and independent variables, which was selected based on their clinical or statistical significance at the univariate analysis. The dependent variable was defined using the item “Would you be willing to donate your body after death?”, classified as “No” and “Yes.” The Hosmer‐Lemeshow (HL) test was used to assess the goodness–of‐fit of the model (a *p*‐value of >0.05 indicates that the model fits the data adequately). A two‐tailed *p*‐value less than 0.05 was used as statistically significant; statistical analysis was performed with STATA version 17 software.

## RESULTS

### Participants characteristics

A total of 440 subjects took part in the web survey, six of them withheld their consent to participate (19.1% response rate, *n* = 2273). Among 434 respondents, the median (IQR) age was 23 (21–31) years, with 70.1% female, 88.0% students, and 12.0% academic staff. Overall, 53.7% were religious believers (Table 1).

**TABLE 1:** Participants characteristics.

Characteristics	Sample (*n* = 434)
Median (IQR) age, years	23 (21–31)
Females, *n* (%)	304 (70.1)
Academic status, *n* (%)	
Students	382 (88)
Academic staff (professors and research fellow)	52 (12)
Study course, *n* (%)	
Medicine and Surgery/Dentistry	173 (45.3)
Health profession MSc	9 (2.4)
Health profession BSc	144 (37.7)
Post graduate education	56 (14.7)
Age by academic status	
Students of BSc and MSc, median (IQR)	22 (20–25)
Students of postgraduate education, median (IQR)	31 (29.0–34.5)
Academic staff, mean (SD)	49.5 (11.2)
Year of study course attended *n* (%)	
I	95 (25.8)
II	102 (27.7)
III	119 (32.3)
IV	28 (7.6)
V	9 (2.5)
IV	15 (4.1)
Religious beliefs, *n* (%)	
Non‐believer	201 (46.3)
Non‐practicing believers	175 (40.3)
Practicing believers	58 (13.4)
Type of religious beliefs, *n* (%)	
Christians	220 (94.4)
Non‐Christians	13 (5.6)
Total family religious faith, *n* (%)	396 (91.2)
Family religious faith, *n* (%)	
Christianity	379 (95.7)
Muslim	3 (0.8)
Mixed	14 (3.5)

### Willingness and attitude toward body donation for education and research purposes

**FIGURE 1:**
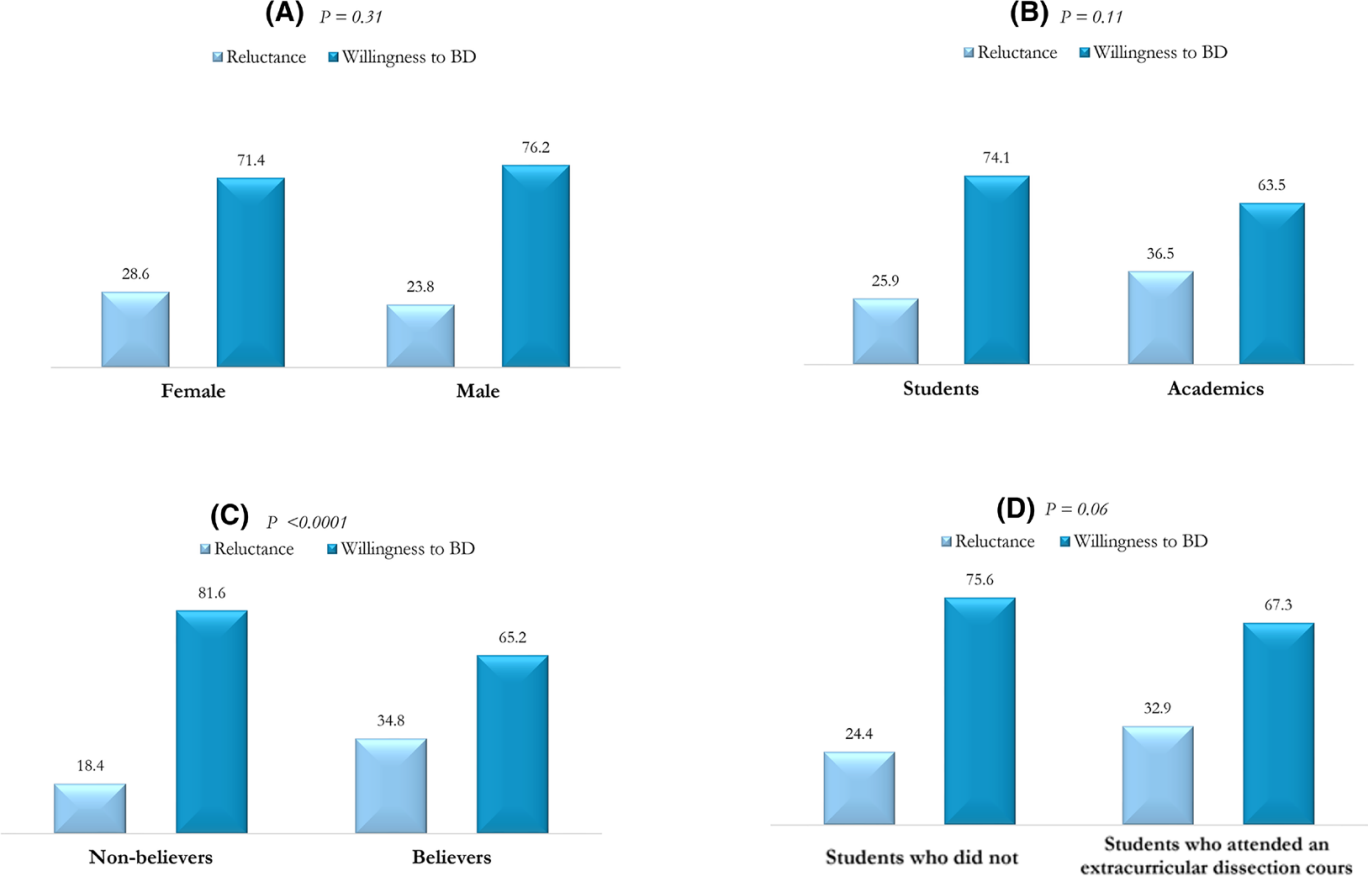
Percentage distribution of willingness to body donation stratified by sex (A), academic status (B), religious beliefs (C), and dissection course (D).

Approximately 72.8% were willing to donate their body, there were no statistical differences based on sex (76.2% and 71.4% in males and females, respectively; *p* = 0.31; Figure 1A) nor academic role (74.1% and 63.5% in students and academic staff; *p* = 0.11; Figure 1B).

The proportion of subjects willing to donate their body was significantly higher in nonbelievers (81.6% vs. 65.2%, respectively; *p* < 0.0001; Figure 1C). Similarly, it was observed that students who did not attend extracurricular dissection training were more willing to donate their own body (75.6% vs. 67.0%; *p* = 0.06) (Figure 1D). As reported in Table 2, more than 70.0% of respondents were willing to donate for educational and/or research purposes. Furthermore, 59.5% declared to know organ donors, 45.2% were blood donors, and 21.7% were involved in social and volunteering activities. The median (IQR) score of the attitude toward body donation as an altruistic, charitable, and solidarity act was 5 (4–5). Similar results were found for body donation considered useful for advancement in scientific research and treatment and as an act of freedom. In contrast, a median score of 1 (1–2) was observed for body donation as an inappropriate act (Tables 2 and S2).

**TABLE 2:** Items questionnaire about willingness and attitudes toward body donation.

Willingness and attitudes toward body donation	Sample (*n* = 434)
Would you be willing to donate your body after death? *n* (%)	
No	118 (27.2)
Yes, for research purposes only	70 (16.1)
Yes, for educational (e.g., dissection) and training purposes	11 (2.5)
Yes, both for research and educational purposes	235 (54.2)
If your response to the above question was “no,” please specify a reason, *n* (%)	
It is in contrast to my religion	2 (1.7)
It is in contrast to the opinion of my family (close people)	28 (23.7)
It is inappropriate as a violation of body	8 (6.8)
It causes me anxiety	58 (49.2)
I would prefer cremation*	7 (5.9)
I don't know, I need more time*	7 (5.9)
I would prefer donate organ to save other lives*	8 (6.8)
Do you know any organ donors? Yes, *n* (%)	258 (59.5)
Are you a blood donor? Yes, *n* (%)	196 (45.2)
Are you currently involved in social work through volunteer activities? Yes, *n* (%)	94 (21.7)
Please, indicate how much you agree or disagree with the following statement: Median (IQR), scores (range from 1—completely disagree to 5—completely agree)	
Body donation is an act of charitable/altruism/solidarity	5 (4–5)
Body donation is helpful for advance in medical research	5 (4–5)
Body donation is an act of freedom	5 (4–5)
Body donation is an inappropriate	1 (1–2)

* Relative to “other” categories.

### Knowledge of national law on body donation

The majority of participants (68.0%) were unaware of the national law regulating body donation for scientific purposes (Table 3). Among those who were aware (*n* = 139, 32.0%), the primary sources of information were mass and social media (45.3%), scientific literature (30.9%), university settings (5.8%), and the workplace (5.0%) (Table 3). No significant differences were found between students and academics, with a similar proportion of individuals aware of the national law in both groups (31.4% vs. 36.5%, respectively, *p* = 0.46).

**TABLE 3:** Items questionnaire about legislative framework and dissection practice.

Legislative framework and dissection practice	Sample (*n* = 434)
Do you know law 10 February 2020 regulating body donation for scientific purposes? *n* (% Yes)	139 (32)
Source of information about Law. 10/02/2020, *n* (%)	
Workplace/colleagues	18 (13.0)
Friends/family	7 (5.0)
Mass and social media	63 (45.3)
Scientific literature	43 (30.9)
University setting, lessons	8 (5.8)
Are there any dissection practices in your degree course/specialist training/workplace? *n* (%)	
No	222 (51.2)
Yes	145 (33.4)
I do not know	67 (15.4)
Would you participate in dissection training? *n* (%)	408 (94.0)
If “no,” please specify your reason, *n* (%)	
The idea of human dissection makes me emotional	6 (23.1)
Lack of interest/Unrelated with my filed/not relevant for my study course	20 (76.9)
Do you believe that participating in practical training courses involving body donors and/or tissue could generate anxiety? Yes—*n* (%)	135 (31.1)
Have you attended extracurricular dissection training? *n* (%)	
No	291 (67.0)
Yes, at my university	120 (27.7)
Yes, at another University	23 (5.3)
If “yes,” please specify where did you attended it, *n* (%)	
Italy	14/23 (60.1)
Abroad	9/23 (39)

### Body dissection practices and related anxiety

A large majority (94.0%) of participants would be willing to participate in anatomy education using dissection, 27.7% attended extra‐curricular body dissection training at their own university, and 5.3% in another university. Whereas 31.1% of participants stated that dissection practice might generate anxiety.

### Willingness to donate and related factors

For some characteristics of interest, we evaluated the differences between participants based on the primary study outcome, that is, willingness to donate their body. Among those unwilling to donate their body, a higher proportion held religious beliefs (68.6% vs. 48.1%, *p* < 0.0001) (Table S3). No differences were found stratifying by religious beliefs of their family. Among participants willing to donate their own body, the proportion who declared to know organ donors was significantly higher (64.2% vs. 46.6%, respectively; *p* = 0.001, Table S3).

**FIGURE 2:**
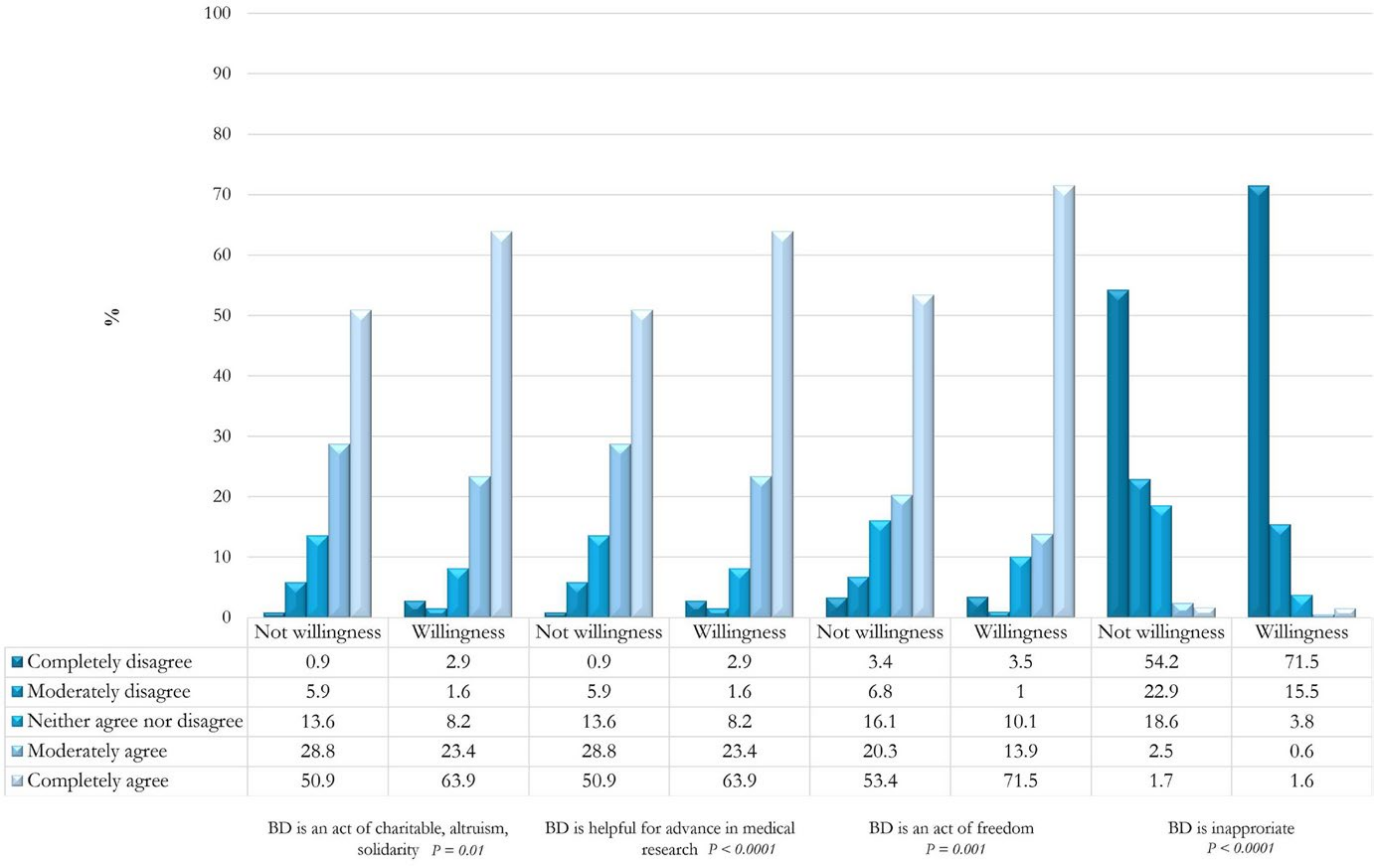
Attitudes toward body donation. The bar chart displays the distribution of ratings on a 5‐point Likert scale for two distinct groups (reluctance to body donation and willingness to body donation). Each column represents one of the five possible ratings (from Completely disagree to Completely agree), and the different sections within the columns indicate the relative percentages for each of the stratifications considered. Box under graph shows percentage values for each response. The *p*‐value refers to the comparison between the two groups for each domain analyzed (i.e., BD is an act of charitable, altruism, and solidarity; BD is helpful for advance in medical research; BD is an act of freedom; BD is inappropriate).

Additionally, a higher proportion of those unwilling to donate reported that body dissection might generate anxiety. Both groups recognize body donation as an altruistic and freedom act as well as a useful action for advances in scientific research and treatments (Figure 2).

### Multivariate analysis

**FIGURE 3:**
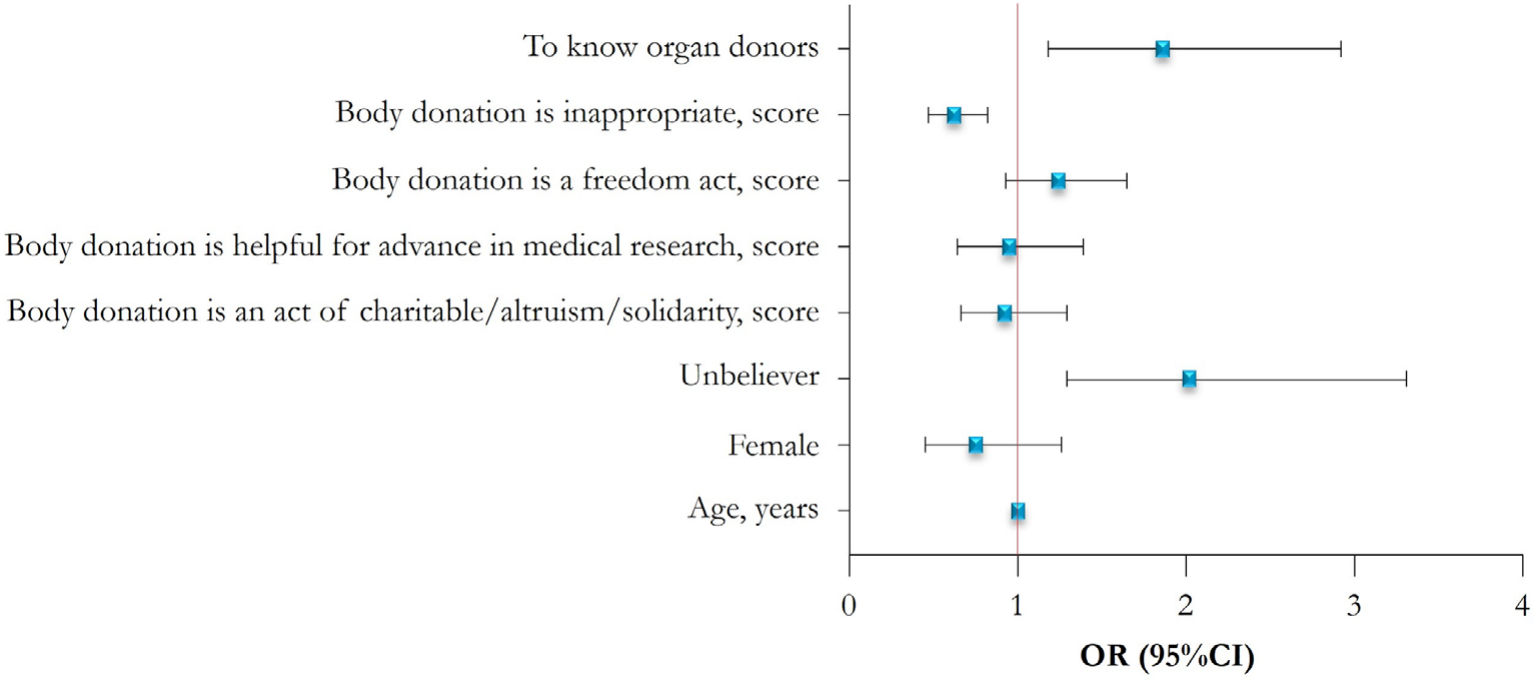
Multivariate analysis: Factors associated with the willingness to donate one's body. Forest plot showing the result of multivariate logistic regression analysis. The *x*‐line represents odds ratio (blue squares) and 95% confidence intervals (whiskers). The red line indicates no effect (*p* > 0.05).

Multivariate analysis revealed factors significantly associated with the willingness to donate one's body: knowing organ donors, perceiving body donation as inappropriate, and religious beliefs (Figure 3).

Specifically, knowing organ donors [OR (95% CI) = 1.86 (1.18–2.92); *p* = 0.007] and being a nonbeliever [OR (95% CI) = 2.01 (1.28–3.30); *p* = 0.003] were positively associated with willingness to donate their body. Otherwise, considering body donation as an appropriate act, measured on a scale from 1 to 5 where higher scores indicate a stronger perception of inappropriateness, was negatively associated with a willingness to donate one's body [OR (95% CI) = 0.62 (0.47–0.82); *p* = 0.001] (Figure 3 and Table S4). The Hosmer‐Lemeshow test *p*‐value was 0.16.

## DISCUSSION

This study provides insights into the willingness to donate one's body for education and research purposes among members of an Italian University, alongside an exploration of their knowledge and sources of information regarding the relevant legislative frameworks.

The main study findings revealed a generally positive attitude toward body donation within the study population. Sociodemographic factors, such as sex and age, as well as academic status (student vs. academic staff) did not significantly influence willingness to donate. Instead, religious factors emerged as key determinants. Additionally, a notable gap in knowledge and awareness regarding legislative aspects related to body donation was identified.

### Academic and students' attitudes

Academic staff showed a positive attitude, in line with findings from Quiroga‐Garza et al.^53^, who reported that the majority of professors supported body donation. This result may stem from the belief that donor dissection is essential for anatomical understanding.^11^ Interestingly, the positive support was higher in our study compared to those conducted in Nigeria,^54^ the Netherlands,^55^ and Spain.^7^, ^56^, ^57^ Variations in teaching experience or cultural attitudes could explain these differences. While overall support for body donation was high among academics, it is important to note that a significant proportion—over 30%—expressed a lack of support. Although this difference compared to students was not statistically significant, the proportionally higher reluctance among academics warrants attention. This may reflect underlying ethical concerns, varying degrees of exposure to body dissection, or differing perspectives on the acceptability of body donation for educational purposes. These findings suggest the need for targeted educational and ethical discussions, even within academic communities, to address persistent reservations.

Similarly, most students enrolled in this study expressed a strong willingness to donate, in line with previous studies.^43^, ^53^, ^58^, ^59^ Comparable results have also been reported among nursing and physiotherapy students, who were generally in favor of body donation for scientific research purposes (Likas et al., 2023).^60^ The predominance of females respondents align with established trends in biomedical sciences across Italy.^61^ The inclusion of students from a range of health sciences courses may explain the slightly lower percentage of willingness compared to Ciliberti et al.^42^, who focused solely on medical students. This suggests that perception of body donation may vary between medical and nonmedical students as also demonstrated in Bolcato et al.'s study.^43^

A notable finding was the substantial support for body donation for “*research and educational purposes*,” compared to “*educational (e.g., dissection) and training purposes*.” One plausible explanation is that including the term “dissection” might have deterred some respondents due to discomfort or negative associations.^5^, ^62^, ^63^ Additionally, “*research and educational purposes*” may be perceived as contributing more broadly to social benefits.

However, this preference may also reflect a widespread misunderstanding among potential donors regarding how donated bodies are used. The vast majority of donated bodies are employed for anatomical education and training, rather than for medical research, and donors typically do not have the option to specify the use of their body.^64^ This misconception may have influenced participants' responses, particularly the high number who selected *advancing medical research* as the primary motivation for donation. This highlights a critical need for improved donor education to clarify how donations are utilized.

Furthermore, the overlap between the two response options—*research and educational purposes* and *educational and training purposes*—combined with the requirement to select only one, may have further confounded participants' choices. This finding suggests that terminology plays a crucial role in shaping public perceptions and attitudes toward body donation. The way concepts such as “donation,” “education,” and “research” are framed can influence not only understanding but also willingness to participate. Therefore, educational campaigns must carefully consider their wording to promote accurate comprehension and engagement.

However, even when individuals express positive views about the importance of body donation, this does not always translate into actual willingness to donate. The gap between acknowledging the value of body donation and committing to it personally has been documented in several studies. For example, a study in the United Arab Emirates (UAE) found that while 86% of university members—including students, professors, and staff—recognized the importance of body donation, only 29% were willing to donate their own bodies.^51^ Similarly, research from a Turkish University study reported that although 64.2% of respondents acknowledged the utility of body donation for medical education, only 26.2% expressed a willingness to donate.^65^

Our findings reflect a similar pattern. In our study, 72.8% of participants indicated a willingness to donate their bodies; however, various barriers, such as religious beliefs and dissection‐related anxiety may hinder actual follow‐through. These obstacles reflect patterns observed in other countries, indicating that despite broad support in principle, personal hesitations often undermine actual commitment. The Italian context may further evolve under the influence of the new national law, which clearly affirms the primacy of the donor's autonomous decision—potentially reducing family‐based refusals and reinforcing respect for individual choice.

Conversely, some countries have implemented culturally sensitive approaches to overcome these barriers. In Taiwan, for example, initial resistance to body donation was mitigated by the Tzu Chi Foundation's emphasis on altruism and the honoring of donors as “silent mentors.” This strategy, supported by public memorial services, has led to more than 40,000 pledges since 1994.^66^ Such examples underscore the power of culturally tailored messaging and institutional commitment in shaping public attitudes.

In addition to emotional or cultural concerns, the mismatch—frequently observed between positive attitudes and actual willingness to donate—may also be driven by practical gaps in knowledge. Sahin et al.^67^ described how a lack of information about the donation application process and where to donate can prevent even motivated individuals from following through. Addressing both informational and emotional barriers is thus essential to narrowing the gap between intention and action in body donation.

Effective communication initiatives, such as “Donate for Science” documentary in New Zealand, can significantly boost donor rates.^68^ On the other hand, a lack of information may lead to fewer body donations,^69^ and adverse publicity can hinder donations.^70^

In our study, mass and social media were the main sources of information for 45.3% of respondents, consistent with previous findings.^71^, ^72^, ^73^ In contrast, only 5.8% reported receiving information through university channels, highlighting a significant institutional gap in raising awareness about the legislative framework for body donation.

Although our study did not specifically explore where participants gained knowledge about body donation in general, we did assess awareness of the new Italian law. This law introduces a significant ethical shift by explicitly affirming the primacy of the donor's decision over family objections. Thus, it is not merely a question of knowing how to donate, but of recognizing the legal and ethical weight of individual autonomy in postmortem decisions.

Despite this limited legal knowledge, willingness to donate was not significantly affected among our healthcare population, likely because they already recognize the intrinsic value of body donation. However, promoting legal awareness remains important, as previous studies have shown that informed individuals are generally more favorable toward donation.^43^ This lack of awareness, particularly regarding the inability of family members to override the donor's consent, could hinder the law's effectiveness. Without widespread understanding of this critical change, individuals may still feel compelled to consult or defer to their families, thereby perpetuating barriers the law was intended to remove.

Increased support and advocacy from healthcare professionals may positively influence public attitudes, as demonstrated by Şahin et al.^67^ To sustain and strengthen donation rates, it is essential to provide clear, accurate information about body donation procedures, relevant legislation, and the respectful handling of donors.^65^, ^74^

### Emotional and psychological factors

Emotional and psychological responses to body donation and dissection must be considered.^75^ Exposure to deceased bodies can trigger emotional distress,^42^, ^76^, ^77^ although most students adapt over time.^5^, ^78^, ^79^, ^80^, ^81^, ^82^, ^83^

Our survey found that students who attended dissection courses were less inclined to donate, confirming findings from previous studies.^84^, ^85^, ^86^ This may reflect negative emotional reactions, particularly among female students.^5^, ^62^, ^63^, ^87^, ^88^ At the same time, the absence of dissection courses at our university may contribute to idealized perceptions of body donation, potentially enhancing support for it among students unfamiliar with the practical realities of anatomical education.

Participants unwilling to donate reported slightly higher anxiety levels, aligning with the existing literature indicating that lower death‐related anxiety is associated with greater willingness to donate.^84^, ^89^ Such anxiety may be exacerbated by negative perceptions of dissection environments. Therefore, improving dissection room conditions–such as enhancing ventilation and managing odors–could help reduce discomfort and lower psychological barriers to donate.^34^, ^90^

In addition to emotional and informational barriers, concerns regarding the potential for disrespectful handling of donated bodies must also be carefully addressed, as such apprehensions can significantly influence individuals' attitudes toward body donation.^91^ Enhancing health literacy and implementing targeted educational initiatives have been shown to positively influence public attitudes and behaviors in this context.^92^, ^93^, ^94^ Furthermore, it is essential to inform the public that, beyond complying with the formal guidelines established by anatomical associations, the anatomical community is guided by a strong ethical commitment of respect and dignity toward individuals who altruistically donate their bodies.^95^ The new Italian Law further advances this framework by legally affirming the primacy of the donor's will over familial objections, reinforcing donor autonomy and aligning legal standards with ethical best practices.

### Sociocultural and religious factors

Age, gender, and religion all influence body donation attitudes.^96^, ^97^, ^98^ Although earlier studies reported mixed gender‐related findings,^22^, ^46^, ^51^, ^89^, ^91^, ^99^, ^100^ our study, consistent with others,^101^, ^102^, ^103^ found no significant associations with sex, age, or academic status.

In contrast, religion emerged as a significant factor influencing willingness to donate. Nonbelievers were more willing to donate, consistent with previous research.^42^, ^51^, ^53^, ^65^ Although religious beliefs are often cited as a reason for reluctance, leaders of major faiths—including Buddhism, Hinduism, Islam, and Christianity—generally support donation.^49^, ^51^, ^65^, ^102^, ^104^, ^105^, ^106^ Both religious belief and atheism influence perceptions of life and death, shaping attitudes toward postmortem donation.^53^, ^107^, ^108^, ^109^, ^110^ The prevalence of nonbelievers and nonpractising believers in our sample reflects broader secularization trends in Italy, particularly among younger generations [ISTAT data, http://dati.istat.it/index.aspx?queryid=24347]. This is consistent with broader evidence indicating that medical and health profession students are less likely to actively practice religion, with some studies also suggesting a gradual detachment from religious practice during medical training.^111^, ^112^, ^113^ While these studies do not always distinguish between nonbelievers and nonpracticing believers, they support a general trend toward lower religious involvement in healthcare professions, potentially influenced by scientific education and professional socialization.

### Altruism and Organ donation influence

Other key findings from our study highlight the strong and positive attitudes toward body donation, which is widely regarded as a charitable, altruistic, and autonomous act. This perception reflects a broader recognition of body donation as a meaningful contribution to advancing medical science and education. Such motivations are consistent with those observed in a multicenter study across Ireland, New Zealand, and South Africa, where support for science and education emerged as primary drivers of donation.^89^ However, as shown in other contexts, altruism alone does not fully explain donation behavior. In Thailand, for instance, while many individuals expressed altruistic motivations, a significant increase in pledges followed the donation of a revered Buddhist monk—illustrating the powerful influence of cultural and religious factors.^114^ These examples underscore the complexity of donor motivation, which often combines altruistic intent with personal, spiritual, or cultural meaning, as suggested by Bolt et al.^115^ Despite recognizing the value of donation, a notable proportion of participants in our study (30%) expressed unwillingness to donate their own bodies while still acknowledging body donation as an altruistic act. This discrepancy reflects a broader phenomenon often observed in the literature, where individuals support the concept of body donation in principle but hesitate to apply it personally or to their relatives.^45^, ^49^, ^85^ Such ambivalence may stem from emotional discomfort, cultural beliefs, or a limited understanding of the donation process. Additionally, our findings show that willingness to donate was significantly influenced by familiarity with organ donation—whether through knowing someone who donated or received an organ—underscoring the role of experiential knowledge in shaping attitudes.^98^ These insights suggest that increasing public exposure to real‐life donation stories and improving communication around the donation process may reduce hesitation and enhance willingness to donate.

It is important to note that our cohort consisted of medical students and academics, whose background in health sciences may shape their views on body donation. While one might expect that increased scientific training fosters greater appreciation for body donation, our findings suggest a more complex dynamic. Notably, most students in the sample had not yet participated in cadaveric dissection. This limited direct exposure may have influenced their positive attitudes, as body donation remained a conceptual and altruistic ideal rather than a tangible experience. Interestingly, students who had undergone dissection were somewhat less inclined to support body donation, a trend also reported in other studies.^84^, ^85^, ^86^ This suggests that first‐hand experience with cadavers may evoke emotional, ethical, or psychological responses that complicate previously held views about donation. Thus, familiarity with medical and research contexts alone does not uniformly translate into greater willingness to donate; the nature and depth of that exposure are equally important in shaping perceptions.

### Low response rate

Despite the valuable findings of this study, the low response rate must be acknowledged. Low response rate is a well‐documented challenge in survey‐based research, particularly among student populations and within academic environments.^116^, ^117^ Several factors likely contributed to this outcome. The survey was distributed during a period of high academic activity, which may have affected participants' availability and motivation to respond. Although we aimed to balance the amount of information collected with the time required to complete the questionnaire, it may have been perceived as too lengthy, posing a barrier for some respondents. Moreover, while the study was presented as an academic research project, participants received no specific counseling regarding its benefits. Despite the anonymity typically associated with higher response rates, the absence of explicit motivation may not have been sufficient in this case.

The response rate we observed (19.4%) is in line with previous studies reporting similar outcomes in email‐based surveys^116^, ^118^ and with those recorded in studies on body donation among university students. For instance, similar rates were reported in an Italian university‐based survey,^43^ while studies reporting slightly higher response rates^53^, ^66^ often relied on active promotion or in‐person recruitment strategies, which were not employed in our study.

Although a high response rate is generally desirable, a lower rate does not inherently invalidate study findings when appropriate methodological safeguards are applied.^119^ The final sample size was determined by a priori power analysis and was sufficient to meet the study's objectives. Furthermore, the confidence intervals around key outcomes do not show extreme variability, suggesting that the findings are robust and unlikely to be the result of random variation. Thus, it might provide meaningful insights into the factors influencing willingness to donate one's body.

### Limitations of this study

The study findings should be interpreted considering some limitations, including the cross‐sectional design of the study which relies on self‐reported data and the overall low response rate, which may have introduced response bias. Other limitations are related to the use of a nonvalidated questionnaire and that fact that the survey was restricted to one university alone. Additionally, this study did not collect some potential unobserved confounding factors related to consent and organ donation policies.

Furthermore, the sample primarily consisted of young university students, predominantly female, with a background in medical and health sciences. While this demographic reflects established trends in biomedical and health‐related degree programs in Italy,^61^ it does not represent the broader population of body donors, who are typically over the age of 60.^98^ Therefore, the attitudes observed in this study may differ significantly from those in the general community.

Additionally, a large proportion of respondents reported knowing an organ donor. However, the survey did not distinguish between knowing someone who is registered as a donor versus someone who has actually donated organs, either as a living or a deceased donor. This ambiguity limits the interpretation of the influence of personal connection to organ donation on participants' attitudes.

Moreover, while our participants are likely more familiar with the educational value of body donation due to their academic training, the cohort consisted predominantly of students who had not yet participated in anatomical dissection. This lack of hands‐on experience may have shaped their perspectives in a more idealized or theoretical manner.

Future research should involve a broader and more diverse population, including individuals from different age groups, educational backgrounds, and regions of Italy, to better assess public attitudes and motivations toward body donation.

## FUTURE PERSPECTIVES

The findings of this study offer valuable insights into the willingness to donate one's body for research and education, particularly within institutions training future public health professionals. These results can guide the development of policies and educational strategies aimed at increasing awareness and addressing misconceptions about body donation. In the context of recent legal changes in Italy, this evidence can support targeted communication initiatives and help clarify the ethical and legal frameworks surrounding donation. The study also identifies knowledge gaps and barriers among students and academic staff, which can inform future educational campaigns. While limited to a single institution, the findings highlight the need for broader, nationwide research and suggest that longitudinal studies could track changes in attitudes over time. Overall, this research contributes to a more informed public discussion and offers guidance for strengthening and expanding ethically sound body donation programs.

## CONCLUSION

This study highlights strong support for body donation among healthcare students and academics, who largely perceive it as an altruistic and meaningful contribution to medical science. However, despite this favorable attitude, the study revealed notable gaps in knowledge about the current legislative framework, even among individuals with a healthcare background.

These findings have significant implications for educational strategies and policy development—especially in Italy, where the legal framework for body donation is relatively recent. Academic institutions should take a more active role in advocacy and curriculum design to ensure that both healthcare professionals and the broader public are informed about the ethical, legal, and practical aspects of donation.

Although the study's response rate was limited and further research is warranted to validate and expand upon these findings, this work contributes a critical perspective to the evolving discourse on body donation.

## AUTHOR CONTRIBUTIONS

**Mariangela V. Puci:** Conceptualization; data curation; formal analysis; investigation; methodology; project administration; writing – original draft; writing – review and editing. **Maria A. Sotgiu:** Conceptualization; investigation; methodology; project administration; writing – original draft; writing – review and editing. **Narcisa Muresu:** Writing – original draft; writing – review and editing. **Laura Saderi:** Data curation; formal analysis; writing – review and editing. **Veronica Moretti:** Writing – review and editing. **Stefano Ratti:** Writing – review and editing. **Andrea F. Piana:** Writing – review and editing. **Andrea Montella:** Project administration; writing – review and editing. **Giovanni Sotgiu:** Conceptualization; data curation; formal analysis; investigation; methodology; project administration; supervision; writing – original draft; writing – review and editing.

## FUNDING INFORMATION

This research received no external funding.

## CONFLICT OF INTEREST STATEMENT

The authors declare no conflict of interest.

## ETHICS STATEMENT

The University Ethical Committee approved the study protocol (n. 8506, February 2, 2023).

## INFORMED CONSENT STATEMENT

Informed consent was obtained from all subjects involved in the study.

## Supporting information


**Table S1.** Survey questionnaire.

**Table S2.** Attitudes toward body donation.

**Table S3.** Sociodemographic characteristics and attitudes between willingness and reluctance to body donation.

**Table S4.** Univariate and multivariate analyses.

**Table S5.** Italian version questionnaire.

## Data Availability

The data that support the findings of this study are available from the corresponding author upon reasonable request.
