# Supplementary material for: Body donation under Italy's recent legal reforms: A cross‐sectional study of attitudes, beliefs, and educational gaps among medical students and faculty
Source: Anat Sci Educ. 2025 Jul 6;18(9):923–36. doi: 10.1002/ase.70084 (PMC12413481; doi:10.1002/ase.70084)
Supplement: Supplementary file 1 — Table S1. Survey questionnaire. [file ASE-18-923-s003.docx]

**Supplementary material**

**Table S1.** Questionnaire

| **SOCIODEMOGRAPHIC** |
| --- |
| **Sex**   - Male - Female |
| **Age (years)** |
| **Current position (academic status)**   - Students (including PhD and Medical specialization) - Professors - Scientist / Researcher |
| **Study course**   - School of Medicine - Dentistry - Nurses - Midwifery - Health professions for rehabilitation - Technical health professions - Health professions for preventive care - Health Professions Sciences (MSc) - Post-Laurea (Master, PhD, medical specialisation) |
| **Year of study course attended** |
| **Religious beliefs**   - Non-believer - Non-practicing believer - practicing believer |
| **Religious faith**   - None - Christianity - Muslim - Buddhism - Hinduism - Other: please specify |
| **Family religious faith**   - None - Christianity - Muslim - Buddhism - Hinduism - Other: please specify |
| **WILLINGNESS, ATTITUDES, AND KNOWLEDGE** |
| **Do you know law 10 February 2020 regulating body donation for scientific purposes?**   - Yes - No |
| **If “yes”, please specify source of information about** **Law. 10/02/2020:**   - Workplace / colleagues - Friends / family - Mass and social media - Scientific literature - University setting, lessons - Other: please specify |
| **Are there any dissection practices in your degree course/specialist training/workplace?**   - Yes - No - I don’t know |
| **Would you participate in dissection training?**   - Yes - No |
| **If “no”, please specify your reason:** |
| **Do you believe that participating in practical training courses involving body donors and/or tissue could generate anxiety?**   - Yes - No |
| **Have you attended extracurricular dissection training?**   - Yes, at my university - Yes, at another university - No |
| **If “yes”, please specify where did you attended it:** |
| **Would you be willing to donate your body after death?**   - No - Yes, for research purposes only - Yes, for educational (e.g., dissection) and training purposes - Yes, both for research and educational purposes |
| **If “no”, please specify your reason:**   - It is in contrast to my religion - It is in contrast to the opinion of my family (close people) - It is inappropriate as a violation of body - It causes me anxiety - Other: please specify |
| **Please, indicate how much you agree or disagree with the following statement:**  (range from 1- completely disagree to 5- completely agree)   \|  \| **1.Completely**  **disagree** \| **2.Moderatly disagree** \| **3.Neither agree nor disagree** \| **4.Moderatly agree** \| **5.Completely agree** \| \| --- \| --- \| --- \| --- \| --- \| --- \| \| Body donation is an act of charitable, altruism, solidarity \| **O** \| **O** \| **O** \| **O** \| **O** \| \| Body donation is helpful for advance in medical research \| **O** \| **O** \| **O** \| **O** \| **O** \| \| Body donation is an act of freedom \| **O** \| **O** \| **O** \| **O** \| **O** \| \| Body donation is inappropriate \| **O** \| **O** \| **O** \| **O** \| **O** \| |
|  |
| **Do you know any organ donors?**   - Yes - No |
| **Are you a blood donor?**   - Yes - No |
| **Are you currently involved in social work through volunteer activities?**   - Yes - No |
