# Supplementary material for: Body donation under Italy's recent legal reforms: A cross‐sectional study of attitudes, beliefs, and educational gaps among medical students and faculty
Source: Anat Sci Educ. 2025 Jul 6;18(9):923–36. doi: 10.1002/ase.70084 (PMC12413481; doi:10.1002/ase.70084)
Supplement: Supplementary file 2 — Table S2. Attitudes toward body donation. [file ASE-18-923-s001.docx]

**Supplementary material: Table S2.**

**Table S2.** Attitudes toward body donation.

| **Attitudes towards body donation** | | **Sample**  **(n= 434)** | **Willingness to body donation** | | **p-value** |
| --- | --- | --- | --- | --- | --- |
|  |  |  | **No**  **(n= 118)** | **Yes**  **(n= 316)** |  |
| *Body donation is an act of charitable/altruism/solidarity, n (%)* | *Completely disagree* | 10 (2.3) | 1 (0.9) | 9 (2.9) | 0.01 |
|  | *Moderately disagree* | 12 (2.8) | 7 (5.9) | 5 (1.6) |  |
|  | *Neither agree nor disagree* | 42 (9.7) | 16 (13.6) | 26 (8.2) |  |
|  | *Moderately agree* | 108 (24.9) | 34 (28.8) | 74 (23.4) |  |
|  | *Completely agree* | 262 (60.4) | 60 (50.9) | 202 (63.9) |  |
| *Body donation is helpful for advance in medical research, n (%)* | *Completely disagree* | 11 (2.5) | 1 (0.9) | 10 (3.2) | <0.0001 |
|  | *Moderately disagree* | 8 (1.8) | 6 (5.1) | 2 (0.6) |  |
|  | *Neither agree nor disagree* | 13 (3.0) | 5 (4.2) | 8 (2.5) |  |
|  | *Moderately agree* | 80 (18.4) | 37 (31.4) | 43 (13.6) |  |
|  | *Completely agree* | 322 (74.2) | 69 (58.5) | 253 (80.1) |  |
| *Body donation is an act of freedom, n (%)* | *Completely disagree* | 15 (3.5) | 4 (3.4) | 11 (3.5) | 0.001 |
|  | *Moderately disagree* | 11 (2.5) | 8 (6.8) | 3 (1.0) |  |
|  | *Neither agree nor disagree* | 51 (11.8) | 19 (16.1) | 32 (10.1) |  |
|  | *Moderately agree* | 68 (15.7) | 24 (20.3) | 44 (13.9) |  |
|  | *Completely agree* | 289 (66.6) | 63 (53.4) | 226 (71.5) |  |
| *Body donation is inaappropriate, n (%)* | *Completely disagree* | 312 (71.9) | 64 (54.2) | 248 (78.5) | <0.0001 |
|  | *Moderately disagree* | 76 (17.5) | 27 (22.9) | 49 (15.5) |  |
|  | *Neither agree nor disagree* | 34 (7.8) | 22 (18.6) | 12 (3.8) |  |
|  | *Moderately agree* | 5 (1.2) | 3 (2.5) | 2 (0.6) |  |
|  | *Completely agree* | 7 (1.6) | 2 (1.7) | 5 (1.6) |  |
