# Supplementary material for: Body donation under Italy's recent legal reforms: A cross‐sectional study of attitudes, beliefs, and educational gaps among medical students and faculty
Source: Anat Sci Educ. 2025 Jul 6;18(9):923–36. doi: 10.1002/ase.70084 (PMC12413481; doi:10.1002/ase.70084)
Supplement: Supplementary file 3 — Table S3. Sociodemographic characteristics and attitudes between willingness and reluctance to body donation. [file ASE-18-923-s004.docx]

**Table S3.** Sociodemographic characteristics and attitudes between willingness and reluctance to body donation.

| **Variables** | | **Willingness toward body donation** | | **p-value** |
| --- | --- | --- | --- | --- |
|  |  | **No**  **(n= 118)** | **Yes**  **(n= 316)** |  |
| *Female, n (%)* | | 87 (73.7) | 217 (68.7) | 0.31 |
| *Median (IQR) age, years* | | 23 (21-33) | 23 (21-31) | 0.67 |
| *Academic status, n (%)* | *Students* | 99 (83.9) | 283 (89.6) | 0.11 |
|  | *Academic staff* | 19 (16.1) | 33 (10.4) |  |
| *Religious beliefs, n (%)* | *Non-believer* | 37 (31.4) | 164 (51.9) | <0.0001 |
|  | *Believer practitioners* | 24 (20.3) | 34 (10.8) |  |
|  | *Believer non- practitioners* | 57 (48.3) | 118 (37.3) |  |
| *Type of religious beliefs, n (%)* | *Non-Christians* | 3 (3.7) | 10 (6.6) | 0.55 |
|  | *Christians* | 78 (96.3) | 142 (93.4) |  |
| *Family believer, n (%)* | | 108 (91.5) | 279 (88.3) | 0.36 |
| *Do you know law 10 February 2020 regulating body donation for scientific purposes? n (%)* | *No* | 83 (70.3) | 212 (67.1) | 0.52 |
|  | *Yes* | 35 (29.7) | 104 (32.9) |  |
| *Would you participate in dissection training? n (%)* | *No* | 10 (8.5) | 16 (5.1) | 0.18 |
|  | *Yes* | 108 (91.5) | 300 (94.9) |  |
| *Do you believe that participating in practical training courses involving body donors and/or tissue could generate anxiety? n (%)* | *No* | 73 (61.9) | 226 (71.5) | 0.05 |
|  | *Yes* | 45 (38.1) | 90 (28.5) |  |
| *Have you attended extracurricular dissection training? n (%)* | *No* | 71 (60.2) | 220 (69.6) | 0.06 |
|  | *Yes* | 47 (39.8) | 96 (30.4) |  |
| *Please, indicate how much you agree or disagree with the following statement:*  *Median (IQR), scores (range from 1- completely disagree to 5- completely agree)* | | | | |
| *Body donation is an act of charitable/altruism/solidarity* | | 5 (4-5) | 5 (4-5) | 0.01 |
| *Body donation is helpful for advance in medical research* | | 5 (4-5) | 5 (5-5) | <0.0001 |
| *Body donation is an act of freedom* | | 5 (3-5) | 5 (4-5) | 0.0003 |
| *Body donation is inappropriate* | | 1 (1-2) | 1 (1-1) | <0.0001 |
| *Do you know any organ donors? Yes, n (%)* | | 55 (46.6) | 203 (64.2) | 0.001 |
| *Are you currently a blood donor? Yes, n (%)* | | 45 (38.1) | 151 (47.8) | 0.07 |
| *Are you currently involved in voluntary social activities? Yes, n (%)* | | 30 (25.4) | 64 (20.3) | 0.25 |

Note: willingness toward body donation (“%Yes”) was defined as all the causes favorable to donation (i.e., for research-, educational or training-, and both conditions purposes).
