# Supplementary material for: Body donation under Italy's recent legal reforms: A cross‐sectional study of attitudes, beliefs, and educational gaps among medical students and faculty
Source: Anat Sci Educ. 2025 Jul 6;18(9):923–36. doi: 10.1002/ase.70084 (PMC12413481; doi:10.1002/ase.70084)
Supplement: Supplementary file 4 — Table S4. Univariate and multivariate analyses. [file ASE-18-923-s005.docx]

**Supplementary material: Table S4.**

**Table S4.** Logistic regression analysis to assess the relationship between survey characteristics and willingness to body donation.

| **Variables** | | **Univariate analysis** | | **Multivariate analysis** | |
| --- | --- | --- | --- | --- | --- |
|  |  | **OR (95% CI)** | **p-value** | **OR (95% CI)** | **p-value** |
| *Females* | | 0.78 (0.49-1.25) | 0.31 | 0.75 (0.50-1.26) | 0.28 |
| *Age, years* | | 0.99 (0.97-1.01) | 0.47 | 1.00 (0.98-1.02) | 0.70 |
| *Academic staff* | *Faculty staff* | Ref. | Ref. | Ref. | Ref. |
|  | *Students* | 1.65 (0.90-3.03) | 0.11 | - | - |
| *Religious beliefs* | *Believer* | Ref. | Ref. | Ref. | Ref. |
|  | *None - unbeliever* | 2.36 (1.51-3.69) | <0.0001 |  | 0.003 |
| *Family believer* | | 0.70 (0.34-1.45) | 0.34 | - | - |
| *Do you know law 10 February 2020 regulating body donation for scientific purposes?* | *No* | Ref. | Ref. | Ref. | Ref. |
|  | *Yes* | 1.16 (0.74-1.84) | 0.52 | - | - |
| *Would you participate in dissection training? Yes* | | 1.74 (0.77-3.94) | 0.19 | - | - |
| *Practice dissection could make you anxious? Yes* | | 0.65 (0.41-1.01) | 0.05 | - | - |
| *Have you attended dissection training independently? Yes* | | 0.66 (0.43-1.02) | 0.06 | - | - |
| *Body donation is an act of charitable/altruism/solidarity** | | 1.26 (1.01-1.56) | 0.04 | 0.92 (0.66 (1.29) | 0.63 |
| *Body donation is helpful for advance in medical research** | | 1.37 (1.09-1.73) | 0.007 | 0.95 (0.64-1.39) | 0.79 |
| *Body donation is a freedom act** | | 1.36 (1.13-1.66) | 0.002 | 1.24 (0.93-1.65) | 0.14 |
| *Body donation is inappropriate** | | 0.56 (0.43-0.71) | <0.0001 | 0.62 (0.47-0.82) | 0.001 |
| *To know organ donor* | | 2.06 (1.34-3.16) | 0.001 | 1.86 (1.18-2.92) | 0.007 |
| *To be currently a blood donor* | | 1.48 (0.96-2.28) | 0.07 | - | - |
| *To be currently involved in voluntary social activities* | | 0.75 (0.45-1.22) | 0.25 | - | - |

*Score range from 1 to 5.

Note: the outcome of the model (i.e., dependent variable) is the willingness to body donation for all reason (0 indicates “no”; 1 indicates “yes”). Model results for independent variables are reported as odds ratios and 95% confidence intervals. In multivariate analysis, odds ratios are adjusted for the effect of each variable included in the model (i.e., gender, age, religious beliefs, body donation considered as an act of charitable/altruism/solidarity, helpful for advance in medical research, freedom-, and inappropriate- act). Hosmer-Lemeshow test p-value= 0.16.
