# Supplementary material for: Body donation under Italy's recent legal reforms: A cross‐sectional study of attitudes, beliefs, and educational gaps among medical students and faculty
Source: Anat Sci Educ. 2025 Jul 6;18(9):923–36. doi: 10.1002/ase.70084 (PMC12413481; doi:10.1002/ase.70084)
Supplement: Supplementary file 5 — Table S5. Italian version questionnaire. [file ASE-18-923-s002.docx]

**Supplementary material: Table S5.**

**Italian version questionnaire**

| ***Informazioni sociodemografiche*** |
| --- |
| **Genere**   - Maschio - Femmina |
| **Età (anni compiuti)** |
| **Posizione attuale**   - Studente (inclusi dottorandi e specializzandi) - Professore - Ricercatore, Borsista / assegnista (esclusi dottorandi e specializzandi) |
| **Se è uno studente che corso di studio frequenta?**   - Medicina e Chirurgia - Odontoiatria - Professioni sanitarie infermieristiche - L/SNT1 (triennale) - Professioni sanitaria ostetrica/o - L/SNT1 (triennale) - Professioni sanitarie della riabilitazione - L/SNT2 (triennale) - Professioni sanitarie tecniche - L/SNT3 (triennale) - Professioni sanitarie della prevenzione - L/SNT4 (triennale) - Laurea Magistrale Professioni sanitarie - Post-Laurea (Master, Dottorato, Scuola di Specialità Medica) |
| **Anno di Corso frequentato** |
| **Si professa credente?**   - Si, praticante - Si, non praticante - No |
| **Credo religioso professato**   - Nessuno - Cristiano cattolico - Cristiano non cattolico - Buddismo - Induismo - Islam - Altro: specificare |
| **Credo della famiglia**   - Nessuno - Cristiano cattolico - Cristiano non cattolico - Buddismo - Induismo - Islam - Altro: specificare |
| ***Informazione, percezione e attitudini*** |
| **Conosce la legge del 10 febbraio 2020 che regolamenta la donazione del cadavere ai fini di studio e di ricerca?** Yes   - Sì - No |
| **Se sì, come ne sei Venuto a conoscenza:**   - Luogo di lavoro / colleghi - Amici/ famiglia - Letteratura scientifica - Media social media - Università, lezioni - Altro: specificare |
| **Nel suo corso di laurea/formazione specialistica/struttura in cui lavora sono previste esercitazioni su tessuto umano cadaverico?**   - Sì - No - Non lo so |
| **Parteciperebbe a corsi di formazione pratica su cadavere e/o tessuto cadaverico?**   - Sì - No |
| **Se ha risposto NO alla domanda precedente, indichi perché non parteciperebbe a corsi di formazione:** |
| **Ritiene che partecipare a corsi di formazione pratica su cadavere e/o tessuto cadaverico potrebbe generare un senso di ansia?**   - Sì - No |
| **Ha partecipato autonomamente a corsi di esercitazione su cadavere e/o tessuti cadaverici?**   - Sì, presso la mia Università - Sì, presso un’altra Università - No |
| **Se ha risposto “Si, presso un’altra Università”, indichi in quale città:** |
| **Sarebbe disposto a donare il suo corpo dopo la morte?**   - No - Sì, per fini di ricerca - Sì, per fini di studio (dissezione) e di formazione - Sì, sia per fini di ricerca che di studio |
| **Se ha risposto no, indichi di seguito una motivazione:**   - Credo sia in contrasto con il mio credo religioso - Credo sia in contrasto con il parere della mia famiglia / delle persone a me care - Credo sia inappropriate in quanto violazione del corpo - L’idea di donare il corpo mi provoca ansia - Altro: specificare |
| **Quanto è d’accordo con le seguenti informazioni:**   \|  \| **1.Completely**  **disagree** \| **2.Moderatly disagree** \| **3.Neither agree nor disagree** \| **4.Moderatly agree** \| **5.Completely agree** \| \| --- \| --- \| --- \| --- \| --- \| --- \| \| La donazione del corpo è un atto di generosità /altruismo/solidarietà \| **O** \| **O** \| **O** \| **O** \| **O** \| \| La donazione del corpo è utile per il progresso della ricerca nell'ambito di cura \| **O** \| **O** \| **O** \| **O** \| **O** \| \| La donazione del corpo è un atto di libertà \| **O** \| **O** \| **O** \| **O** \| **O** \| \| La donazione del corpo è inappropriata \| **O** \| **O** \| **O** \| **O** \| **O** \| |
|  |
| **Conosce dei donatori di organi?**   - Sì - No |
| **E' un donatore di sangue?**   - Sì - No |
| **Attualmente è impegnato nel sociale tramite attività di volontariato?**   - Sì - No |
